# The Effect of Informing Participants of the Response Bias of an Automated Target Recognition System on Trust and Reliance Behavior

**DOI:** 10.1177/00187208211021711

**Published:** 2021-06-02

**Authors:** Shala Knocton, Aren Hunter, Warren Connors, Lori Dithurbide, Heather F. Neyedli

**Affiliations:** 1 3688 Dalhousie University, Halifax, Nova Scotia, Canada; 2 113195 Defence Research and Development Canada, Dartmouth, Nova Scotia, Canada

**Keywords:** human–automation interaction, defense, underwater mine detection, military psychology

## Abstract

**Objective:**

To determine how changing and informing a user of the false alarm (FA) rate of an automated target recognition (ATR) system affects the user’s trust in and reliance on the system and their performance during an underwater mine detection task.

**Background:**

ATR systems are designed to operate using a high sensitivity and a liberal decision criterion to reduce the risk of the ATR system missing a target. A high number of FAs in general may lead to a decrease in operator trust and reliance.

**Methods:**

Participants viewed sonar images and were asked to identify mines in the images. They performed the task without ATR and with ATR at a lower and higher FA rate. The participants were split into two groups—one informed and one uninformed of the changed FA rate. Trust and/or confidence in detecting mines was measured after each block.

**Results:**

When not informed of the FA rate, the FA rate had a significant effect on the participants’ response bias. Participants had greater trust in the system and a more consistent response bias when informed of the FA rate. Sensitivity and confidence were not influenced by disclosure of the FA rate but were significantly worse for the high FA rate condition compared with performance without the ATR.

**Conclusion and application:**

Informing a user of the FA rate of automation may positively influence the level of trust in and reliance on the aid.

Naval mines are low-cost, easily deployed weapons that are difficult to detect, dangerous, and expensive to counter, posing a significant challenge to maritime security (Ho et al., 2011; Pavlovic et al., 2012). Naval mines have been used effectively for decades as a way to control waterspace with widespread impact on commercial and military maritime activities. Many navies have developed mine countermeasures (MCM) capabilities to detect, localize, identify, and neutralize a mine threat. The detection and classification of naval mines uses seafloor visualizations collected with high-frequency sonars mounted on specialized ships or autonomous underwater vehicles (AUVs). Although these sensors provide visualizations with excellent resolution, distinguishing a mine from other mine-sized objects present on the seafloor is still difficult. Modern side-looking sonar systems also generate a substantial amount of data for an operator to parse. To aid operators with analyzing the large volumes of data, automated target recognition (ATR) systems were introduced. For MCMs, ATR algorithms evaluate the side-scan sonar visualizations for objects that have mine-like characteristics. If present, the mine-like object is highlighted for further processing by the operator (Ho et al., 2011; Kessel & Myers, 2005; Myers, 2009).

Though ATR systems have the potential to reduce workload and increase accuracy, the systems typically operate using a high sensitivity and a liberal decision criterion to reduce the risk of missing a mine (parameters of operational systems are not publicly releasable). A liberal decision criterion is often desired in a wide range of ATR tasks, including mine detection, where the cost of missing a target is high. Unfortunately, the number of false alarms (FAs) that occurs as a result tend to lead to a decrease in the trust and reliance/compliance the operators have in the aid (Geels-Blair et al., 2013; Kessel & Myers, 2005; Kessel, 2005; Rice & McCarley, 2011). FAs are more detrimental than misses due to their increased cognitive salience (Rice & McCarley, 2011). In the case where a user is in charge of monitoring both the automation and the raw data, frequent FAs may result in the user having to continuously check the raw data to determine if the alert is correct in identifying a signal as present (Dixon et al., 2007). In addition, a high FA rate may also lead to the “cry-wolf” effect (Breznitz, 1983; Sorkin, 1989), where users ignore the alerts over time, even when they may be correct (Allendoerfer et al., 2008; Parasuraman & Riley, 1997; Satchell, 1993; Sorkin, 1988). Related to the FA rate, the strength of the cry-wolf effect is influenced by the positive predictive value (PPV) of the automation (Huegli et al., 2020; Manzey et al., 2014). The PPV is the probability that a signal is present when the automation says a signal is present. Operator compliance with the automation decreases with lower PPV (Bartlett & McCarley, 2017, 2019; Huegli et al., 2020).

Looking to the broad automation literature, other factors that influence a user’s reliance behavior include trust in automation (Dzindolet et al., 2003; Hoff & Bashir, 2015; Lee & Moray, 1992, 1994; Lee & See, 2004) and user self-confidence (Lee & Moray, 1994; Lewandowsky et al., 2000; Madhavan & Wiegmann, 2004). In circumstances where the error made by the aid is obvious and detected by the user, trust and reliance may be undermined. This may be due to the user believing that they can perform better than the automated system (Madhavan et al., 2003), resulting in the task being completed manually (Dzindolet et al., 2002; Lee & Moray, 1992, 1994; Parasuraman & Riley, 1997). If instead, the user’s trust in the automated system exceeds their confidence in their own abilities, the task tends to be completed using automation. Any changes that may occur in the level of trust the user has in the automation, or confidence in their abilities, is suggested to correspond to an associated change in automation use (Hussein et al., 2020; Lee & Moray, 1994).

Given the high expectations operators often have of automated systems (Ross et al., 2007), properly calibrating operator expectations with information about an automated system’s reliability level tends to result in more appropriate reliance on and monitoring of the aid (Bagheri & Jamieson, 2004). Dzindolet et al. (2003) found that uninformed operators developed unrealistic expectations about the system’s capabilities, whereas operators who were given information regarding the reliability of an automated system were able to develop more appropriate levels of trust in and reliance on the system (Bagheri & Jamieson, 2004; Du et al., 2020; Reiner et al., 2017; Wang et al., 2009). This research shows the benefit of informing operators of the reliability of an automated system, but it does not examine the effect of informing operators of a system’s response bias. Therefore, the present study examines the effect of different response biases and disclosure patterns on the trust, reliance, and performance of users in an underwater mine detection task, a context with a high FA rate. Note that in operationalizing the experiment, we altered the response bias of the ATR, but rather than explaining changing response bias to our participants who may not have been familiar with signal detection theory, we informed participants about the change in the relative rates of FAs and misses. Thus, from here on, we refer to informing the users of the FA rate rather than the response bias.

## Purpose

The purpose of this study was to determine if the FA rate of an ATR system affects a user’s trust in the system, confidence in their own abilities, and mine detection performance during an underwater mine detection task, and whether informing the user of the FA rate of the system affects each of these factors. To this end, two groups of participants performed an underwater mine detection task with the help of an ATR system. The reliability and sensitivity of the aid was held constant across the experimental sessions, but the FA rate of the system changed part way through the study. One group of participants was informed of the change in the FA rate, and one group was not.

## Hypotheses

It was hypothesized that the users’ trust in the automation would be higher during the low FA rate condition compared with the high FA rate condition and that trust would be higher when participants were informed of the automation’s FA rate compared with when they were not informed. Performance was expected to be worse for the high FA rate compared with low FA rate automation. The FA rate set (high vs. low) and whether the participant was informed of the FA rate (informed vs. not informed) were expected to affect participant self-confidence. Due to the limited research on the effect FA rate has on an individual’s confidence, nondirectional hypotheses were used for this measure.

## Methods

### Participants

Seventy adults (age: *M* = 20.13 ± 2.04 years; gender: 55 women, 14 men, 1 no gender specified) with normal or corrected-to-normal vision were recruited from the Dalhousie University community. Each participant provided informed consent, and received credit points for a psychology course and a performance bonus of 10CAD. Participants were told they would only receive the bonus if their mine detection performance was in the top 25%, but all participants received the bonus upon completion of the experiment.

### Stimuli and Measures

#### Stimuli

Participants sat in front of a monitor and interacted with a simulation that displayed sonar images of the seafloor. Participants were tasked with identifying whether a mine was present in the sonar images. To ensure control over the number and type of mines in the images, the mines were synthetically injected into the seafloor images. This procedure was based on a three-step process of sampling and characterizing the environmental noise of the image, applying that noise model to an ideal template of a mine image, and then inserting this noisy target template into the sonar data (Fawcett, 2017). This procedure ensured that physical aspects such as shadow length and specular highlight response were considered, and a reasonable noise distribution to ensure the images appeared correct within the seafloor scene. The participants entered each of their responses by clicking an area in the sonar image where they believed a mine was present or by clicking a black box in the top right-hand corner of the image labeled “NO MINE.”

**Figure 1:**
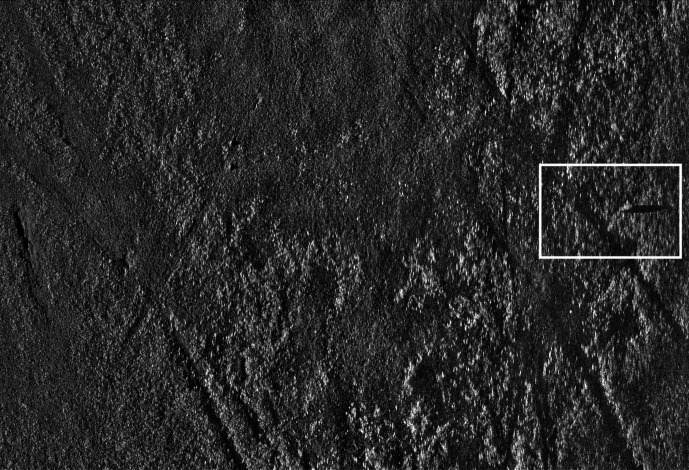
Sonar image with a cue from the ATR system. *Note*. ATR = automated target recognition.

During some blocks of trials, an ATR system aided the participants. For these blocks, a rectangle appeared around a region on the sonar image if the system believed a mine was present (Figure 1).

Participants completed a Trust in Automation Questionnaire (Jian et al., 2000) following each of the experimental blocks where automation was used and a Confidence in Abilities Questionnaire after the training block and each of the experimental blocks. The Confidence in Abilities Questionnaire was an adapted version of the Trust in Automation Questionnaire and was used to assess the participants’ level of confidence in their own abilities to detect underwater mines in the sonar images (see Supplemental Materials for full questionnaires). The coordinates on the images where the participants clicked were recorded using a custom MATLAB script to determine their response. For mouse clicks on the sonar image, if the participants clicked within a 50-pixel square centered on the true mine, it was recorded they had correctly identified a mine (i.e., a hit).

### Design

Participants completed four experimental blocks, two with no automation (50 trials each) and two with automation (100 trials each). In all blocks, a mine was present in half the trials. The use of two no automation blocks allowed us to determine how performance changed over time in our novice participants. These blocks used a fewer number of trials due to the reduced complexity of not having the automated device. In other words, in the automation blocks, there was the added factor of the automation saying the mine was present versus absent overlayed with the mine actually being present or absent, whereas in the no automation blocks the mine was simply present or absent on each trial.

The misses were adjusted for each of the FA rate conditions to ensure that the overall sensitivity (d’ = 2.00) and reliability (83% of the trials had the correct identification) of the system was kept constant, but only the response bias changed. Participants completed one automation experimental block with the low FA rate and the other with the high FA rate. The order of presentation was counterbalanced between participants in each group (informed, not informed). For the low FA rate, 12% of the mine-absent trials had FAs: 6/50 of the trials where a mine was absent had FAs, and 11/50 of the trials where a mine was present had misses. For the high FA rate, 24% of the mine-absent trials had FAs: 12/50 of the trials where a mine was absent had FAs, and 5/50 of the trials where a mine was present had misses.

Participants were randomly assigned to one of two groups. The automated (not informed) group performed the automated experimental blocks without being informed of the FA rate/reliability of the system, and the automated (informed) group performed the automated experimental blocks with being informed of the FA rate/reliability of the system. Participants in the automated (informed) group were informed of the FA percentage via a script (see Supplemental Materials) before starting the trials for the automation experimental blocks.

### Procedure

**Figure 2:**
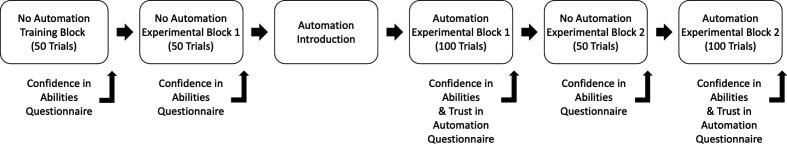
Study design for the training and experimental blocks of trials. One experimental block had high FA automation and the other low FA automation, which was counterbalanced between the participants in each group. *Note*. FA = false alarm.

The participants were told that they would play the role of a naval commander aboard a warship about to sail in unchartered waters with the task of monitoring sonar images collected by an unmanned submarine to identify underwater mines. The participants completed five blocks of trials, one of which was a training block (Figure 2 for study design). The participants received feedback during the no automation training block that indicated whether they were correct in determining if a mine was present or not but did not receive any feedback about their mine detection decisions during the experimental blocks.

During the automation introduction, the participants were informed that an ATR system had been designed to assist them in completing their task. The participants were provided a script explaining that the system uses computer vision to identify the presence of mines but is not entirely perfect and that the system could be altered to change the number of FAs that occur. Participants were then shown example sonar images that contained examples of a hit, miss, correct rejection, and FA by the experimenter.

For the automation experimental blocks, only the participants in the informed group were told whether the FA rate for the experimental block had been set to high or low. The instruction script contained the following text with the text in brackets changing depending on whether the participant was in the high or low FA rate block.

For this mission, your commander has set the sensitivity of the device (high/low). That means that it is expected that (24%/12%) of the trials the automation is going to have a false alarm where it says a mine is present but in fact there is no mine. However, this also means that the system may detect (fewer/more) mines.

All participants were told during the second no automation experimental block that the automation was being re-calibrated and that they would not have access to it for that block of trials.

The participants were asked to complete the Confidence in Abilities Questionnaire following the first training block and each of the experimental blocks, as well as the Trust in Automation Questionnaire only following the automation experimental blocks.

### Data Analysis

Response time was calculated on each trial by taking the difference between when the sonar image appeared on the screen and when the participant clicked the mouse to select a response. Average response time was determined for the trials when a mine was present and was absent.

The proportion of hits and FAs that occurred in each block of trials per participant was calculated by dividing the number of hits and FAs determined by the number of trials in which a mine was present. The hit and FA proportions were then converted to z-scores within MATLAB using an inverse complementary error function and inputted into the following equation to calculate sensitivity:

(1) $$d^{`}=z_{hit}-z_{falsealarm}$$

Using the calculated z-scores for hits and FAs, response bias values were also determined for each block of trials per participant using the equation:

(2) $$\beta =-0.5*(z_{hit}+z_{falsealarm})$$

For the Trust in Automation and Confidence in Abilities Questionnaires, questions were reversed scored as required, and the average Trust and Confidence level was calculated for each questionnaire.

Two approaches were used for analysis. One approach focused on all experimental blocks to detect the difference between the no automation blocks and the two FA rate conditions. This analysis elucidates on the impact of introducing automation and whether there were any training effects by comparing performance in the first and second no automation block. To this end, a 2 (Group: Informed, Not Informed) by 4 (Automation Condition: No Auto 1, No Auto 2, Auto Low FA, Auto High FA) mixed ANOVA was performed on response time when a mine was absent, response time when a mine was present, sensitivity, and confidence. Note that there were no effects of the theoretically relevant manipulations of FA rate or Group on response time, only an effect of block; therefore, the temporal variables are reported in the Supplementary Materials. The second approach focused specifically on the blocks that used the automation to compare the effects of Low and High FA rate on trust in automation and operator response bias; therefore, a 2 (Group: Informed, Not Informed) by 2 (Automation Condition: Auto Low FA, Auto High FA) mixed ANOVA was performed on response bias and trust. For measures where sphericity was violated, Greenhouse–Geisser estimates were used. Effect size, *η_p_*^2^, was calculated for each main effect, and Tukey’s HSD post hoc comparisons were performed to follow up on any main effects involving Automation Condition or significant interactions. Only significant effects are reported.

## Results

### Performance

**Figure 3:**
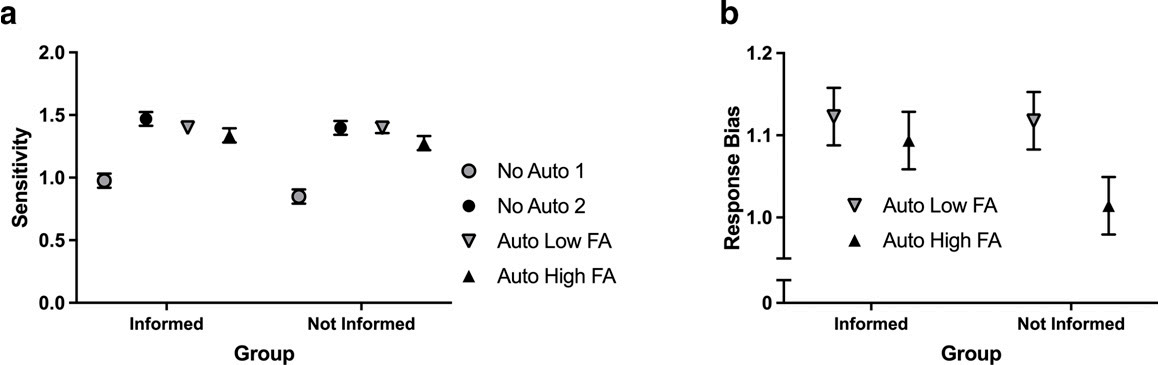
(a) Average sensitivity of participants in the Informed (**I**) and Not Informed (NI) groups for each automation condition, and (b) average response bias of participants per group for the Auto Low FA and Auto High FA conditions. Error bars are standard error of the mean (SEM). *Note*. FA = false alarm.

There was only a significant effect of Automation Condition on sensitivity; *F*(2.60, 176.6) = 68.9, *p* < .01, *η_p_*^2^ = .50 (Figure 3a). Mean sensitivity for No Auto 1 was found to be significantly lower compared with the other three conditions (critical value = .11). In addition, mean sensitivity was also found to be significantly lower in the Auto High FA condition compared with No Auto 2. (Though the use of response bias and sensitivity provide a more nuanced analysis of performance than percentage correct, note that all participants performed better than chance and were on average 70%, 84%, 81%, and 82% accurate in the No Auto 1, No Auto 2, Low FA, and High FA blocks, respectively, mirroring the sensitivity results.)

There was significant main effect of Automation Condition on response bias, *F*(1, 68) = 17.7, *p* < .01, *η_p_*^2^ = .21; however, this effect was superseded by the interaction between Group and Automation Condition, *F*(1, 68) = 5.58, *p* = .021, *η_p_*^2^ = .08 (Figure 3b). In the Not Informed Group, mean response bias was found to be significantly lower (i.e., the participants were more likely to say a mine was present) for the Auto High FA condition (*M* = 1.01, SEM = .04) compared with the Auto Low FA condition (*M* = 1.12, SEM = .04; critical value = .06). However, for the Informed Group, no significant difference in mean response bias was found between the Auto Low FA (*M* = 1.12, SEM = .04) and Auto High FA (*M* = 1.09, SEM = .04) conditions.

### Questionnaires

**Figure 4:**
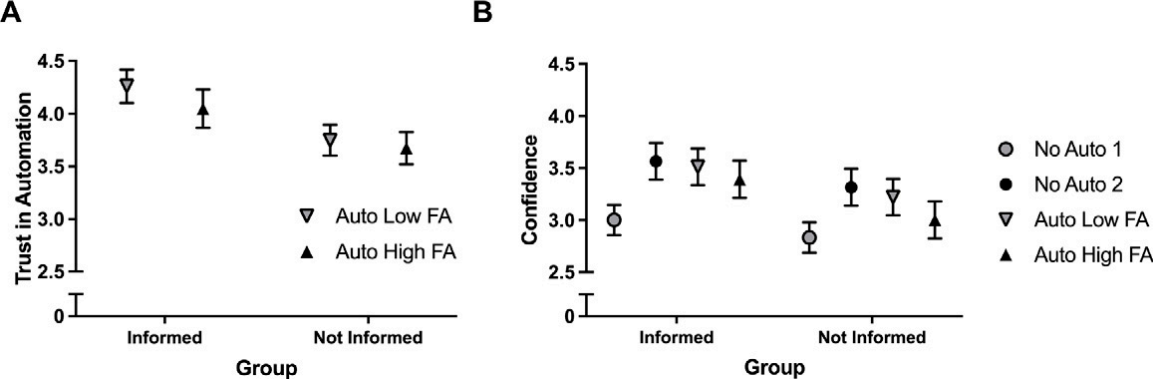
(a) Average trust of participants in the Informed (**I**) and Not Informed (NI) groups for the Auto Low FA and Auto High FA conditions, and (b) average confidence of participants per group for each automation condition. Error bars are SEM. *Note*. FA = false alarm.

Trust in automation was significantly different between the Informed and Not Informed Groups, *F*(1, 68) = 4.37, *p* = .040, *η_p_*^2^ = .06, where the Informed Group had greater trust in the automation compared with the Not Informed Group (Figure 4a). The effects of condition and the interaction were not significant. A significant difference was found in confidence between Automation Conditions, *F*(3, 204) = 24.63, *p* < .01, *η_p_*^2^ = .27, where mean confidence for No Auto 1 was significantly lower compared with the other three conditions (critical value = .17; Figure 4b). In addition, mean confidence was also significantly lower in the Auto High FA condition compared with No Auto 2.

## Discussion

The purpose of this study was to determine if the response bias of an ATR system affects a user’s trust in the system, confidence in their own abilities, and performance during an underwater mine detection task, and whether these variables are influenced by informing the user of the FA rate. Trust in the automation was greater for the participants who were informed of the FA rate compared with the participants who were uninformed of the FA rate. The response bias of the participants in the informed group remained relatively unchanged between the low FA rate and high FA rate automation conditions, where the response bias of uninformed participants was influenced by the system FA rate. Furthermore, sensitivity and confidence levels were lower for the high FA rate condition compared with when no automation was used later in the session (No Auto 2). Participant performance (speed and sensitivity) improved from the first no automation block to the second no automation block, indicating that they still may have been learning about the task during the first block. Therefore, the discussion will mainly focus on comparing the automation conditions with the second no automation block.

### Trust

The finding of increased trust for participants who were informed of the FA rate is consistent with previous research that has indicated that disclosing the reliability level of an automated aid (Hollands & Neyedli, 2011; Neyedli et al., 2011; Wang et al., 2009), or providing information regarding the aid’s behavior or competence (Bagheri & Jamieson, 2004; Lee & See, 2004; Muir, 1994), should lead to more appropriate levels of trust and reliance. Since the participants did not have previous interactions with the ATR system, those who were not informed of the FA rate information may have had to determine the ability of the system by observing its actions and decisions (Bagheri & Jamieson, 2004).

Although informing the participants of the FA rate resulted in an increase in overall trust, there was no difference in trust between the low and high FA rate automation conditions. Trust was expected to be lower in the high FA condition because FA errors may be more cognitively salient than miss errors leading to lower perceived reliability (Rice & McCarley, 2011). According to the literature, as the reliability of an automated aid decreases, a decrease in user trust and performance is expected (Dzindolet et al., 2003; Hollands & Neyedli, 2011; Madhavan et al., 2003; Parasuraman et al., 2000). Since the overall sensitivity of the system and reliability were kept constant for each FA rate condition, this may suggest that trust is less sensitive to changes in an automation’s response bias.

### Participant Response Bias

Participants who were not informed of the FA rate of the automation had a more liberal response bias for the high FA compared with the low FA condition, whereas the response bias for the informed group remained relatively unchanged. These results suggest that when the participants were not informed of the system’s FA rate, their responses were more likely to be influenced by the response bias of the automation, which may be undesirable as the actual base rate of the probability of a mine did not change. This change in user response bias to mirror that of the automation may be attributed to automation bias, or the tendency for users to overly rely on information provided by an automated aid (Dzindolet et al., 2001, 2002; Parasuraman & Manzey, 2010; Parasuraman & Riley, 1997). If a user is aware of the reliability level of automation, they may be more likely to monitor the cues or information generated by the aid appropriately (Bagheri & Jamieson, 2004) and interfere with the system when they do not believe the cue generated is an appropriate response (Colebank, 2008). Furthermore, in situations where automation has a high FA rate, the user may develop a more conservative response bias to compensate for the system’s liberal decision criterion (Wickens et al., 2013). Therefore, informed participants may (1) pay more attention to the cues generated by the automation, (2) be more cautious of the cues in the high FA compared with the low FA rate condition, and (3) intervene when the automated cues deviate from what they believe to be the best response. Over time, these behaviors may improve the performance of the human and automated team.

### Sensitivity and Confidence

The sensitivity and confidence levels of the participants from both groups were found to be lower for the high FA automation condition compared with No Auto 2. According to the literature, when trust in automation exceeds a user’s level of confidence in their own abilities, automation tends to be used, and when a user’s confidence is greater than their trust in the automation, the task tends to be completed manually (Dzindolet et al., 2002; Lee & Moray, 1992, 1994; Parasuraman & Riley, 1997). Therefore, due to the high number of hits performed by the automation during the high FA condition, along with the reported decrease in confidence in their mine detection abilities, the participants may have decided to simply rely on the aid rather than monitor the images and cues appropriately.

When the task was completed manually, the users had similar sensitivity scores and confidence in their own abilities compared with when the task was completed with the low FA automation. Similarly, the users’ detection performance and confidence did not significantly differ between the high and low FA rate conditions. Though there appears to be a small numerical difference between the two groups, with sensitivity and confidence being higher for those in the informed group (Figures 3a and 4b), knowledge of the expected number of FAs did not significantly improve the users’ ability to detect the mines in the images or increase the users’ confidence in their decisions.

### Limitations and Future Research

As with most laboratory experiments, there are potential shortfalls when applying the results to an expert population. Compared with trained sonar operators, the individuals who participated in this experiment were not familiar with ATR systems or the detection of mines. Whereas experienced operators would be quick to identify errors made by the ATR, novices are less likely to identify the same errors. Novices may more easily mistake FAs for hits or misses for correct rejections. Future research should examine the effect FAs have on trained operators who are more likely to identify these errors.

Future research should also investigate the benefit of presenting the PPV of systems that have a liberal decision criterion, such as the case with ATR. Recent work by Huegli et al. (2020) indicates that operators are particularly sensitive to the PPV of detector systems with low base rates. As such, PPV information may better calibrate operator–automation reliance over error rates.

## Conclusion

The purpose of this research was to determine how changing the FA rate of an ATR in a mine detection task affects trust, reliance, and performance and whether informing the user of the FA rate could mitigate the potentially detrimental effects of high FA rates on trust and reliance. When users were not informed of the FA rate of the automation, the number of FAs made by the system affected participants’ reliance behavior and reduced their trust in the system. In this experiment, when the reliability of the automation was kept constant, trust was not affected by the change in response bias. Sensitivity and confidence were not influenced by disclosure of the FA rate, but the performance and confidence measures were significantly worse for the high FA rate condition compared with manual performance. To increase a user’s level of trust in automation and to encourage the user to rely on the recommendations made by the system more appropriately, information should be provided regarding the number of FA errors that are expected to occur. In addition, designers should use caution when implementing systems with a high FA rate due to the detrimental effects it may have on user confidence or performance.

## Key Points

- Automated target recognition (ATR) systems are algorithm-based systems developed to assist or replace human operators in detecting targets of interest.
- The purpose of this study was to determine if the FA rate of an automated target recognition system affects a user’s trust in the system, confidence in their own abilities, and mine detection performance during an underwater mine detection task, and whether informing the user of the FA rate of the system affects each of these factors.
- Two groups of participants performed a simulated underwater mine detection task with and without the help of an ATR system. The reliability and sensitivity of the system was held constant, but the FA rate of the system was set at a low FA rate or a high FA rate and changed part way through the study. One group of participants was informed of the change in FA rate, and one group was not.
- Trust in the automation was greater for the participants who were informed of the FA rate compared with the participants who did not receive any FA rate information.
- Response bias of the participants in the informed group remained relatively unchanged between the low FA rate and high FA rate automation conditions, where the response bias of the participants in the not informed group appeared to be influenced by whether the system was set at a low or a high FA rate.
- Sensitivity and confidence levels were found to be lower for the high FA rate automation condition compared with when no automation was used.
- Informing users that the FA rate of the system can be changed may improve trust by providing more process-based information on how the automation operates.
- Designers should use caution when implementing systems with a high FA rate due to the detrimental effects it may have on user confidence or performance.

## Supplemental Material

Online supplementary file 1 - Supplemental material for The Effect of Informing Participants of the Response Bias of an Automated Target Recognition System on Trust and Reliance Behavior Supplemental material, Online supplementary file 1, for The Effect of Informing Participants of the Response Bias of an Automated Target Recognition System on Trust and Reliance Behavior by Shala Knocton, Aren Hunter, Warren Connors, Lori Dithurbide and Heather F. Neyedli in Human Factors: The Journal of Human Factors and Ergonomics Society
