# Supplementary material for: The Effect of Informing Participants of the Response Bias of an Automated Target Recognition System on Trust and Reliance Behavior
Source: Hum Factors. 2021 Jun 2;65(2):189–99. doi: 10.1177/00187208211021711 (PMC9969489; doi:10.1177/00187208211021711)
Supplement: Online supplementary file 1 - Supplemental material for The Effect of Informing Participants of the Response Bias of an Automated Target Recognition System on Trust and Reliance Behavior [file sj-docx-1-hfs-10.1177_00187208211021711.docx]

**Supplementary Materials: The Effect of Informing Participants of the Response Bias of an Automated Target Recognition System on Trust and Reliance Behaviour**

Knocton, S., Hunter, A., Connors, W., Dithurbide, L., & Neyedli, H.F.

*Human Factors*

**Questionnaires:** The following questionnaires were used in the study (see main text for the time points in the study in which they were delivered). Following each prompt the participant provided their answer on a 7 point Likert scale with the anchors 1- not at all; 7 – extremely.

***Questionnaire about Trust between People and Automation***

1.The system is deceptive

2.The system behaves in an underhanded manner

3. I am suspicious of the system’s intent, actions, or outputs

4. I am wary of the system

5. The system’s actions will have a harmful or injurious outcome

6. I am confident in the system

7. The system provides security

8. The system has integrity

9. The system is dependable

10. The system is reliable

11. I can trust the system

12. I am familiar with the system

***Confidence in Abilities Questionnaire***

**In this section, please answer the questions regarding your confidence in your abilities *WITHOUT THE USE OF THE AUTOMATED SYSTEM.***

1. I believe that my ability to identify mines on my own may lead to negative outcomes.

2. I am not confident in my own ability to identify mines.

3. I am wary of identifying mines on my own.

4. My lack of confidence in my ability to identify mines worsens my performance.

5. I am confident in my ability to identify mines.

6. I feel secure in my ability to identify mines.

7. I feel like others could depend on my ability to identify mines.

8. My ability to identify mines is reliable.

9. I can trust my ability to identify mines.

10. My confidence in my ability to identify mines improves my performance.

**Training and Experimental Scripts:**

Each script was given as a separate sheet before that section of the procedure.

**Mine Detection Training Script**

In this simulation, you will play the role of a naval commander aboard a Navy Frigate. You are about to sail in unchartered waters which could become dangerous due to the possibility of encountering underwater mines. A small unmanned submarine has been released into the waters to capture images of the sea floor. Your task is to examine the images to see if any mines are present. It is important that all mines are identified to ensure safe travels through the water. You must examine all of the images before the ship leaves.

You will complete two training sessions, and four mission blocks that range in length from approximately 5-15 minutes depending on the number of images you have to look at for each mission. We will provide you more information before each mission.

As with other sea floors, rocks, sea grass and debris may be present that could look like mines. Also, enemies will try to disguise the mines to appear like rocks or debris. This could make completing your task very difficult.

Here are some example images. The experimenter will now point out which contain mines and which contain rocks or other things that could be mistaken for mines. After the experimenter goes over this with you, you will have the opportunity to look at 50 different images from a previous mission and indicate whether a mine is present or absent. We will tell you if you were correct.


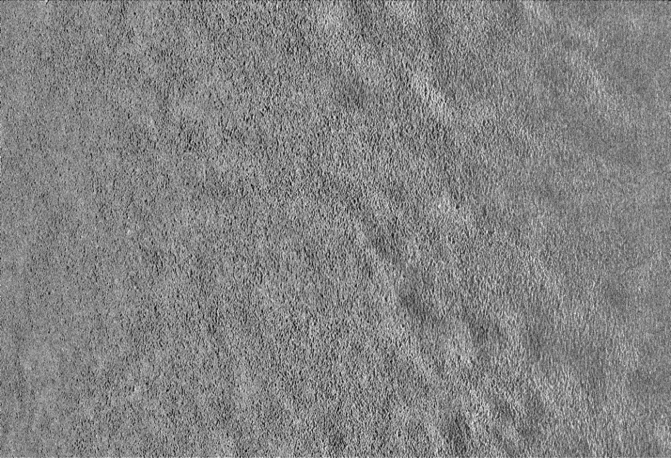

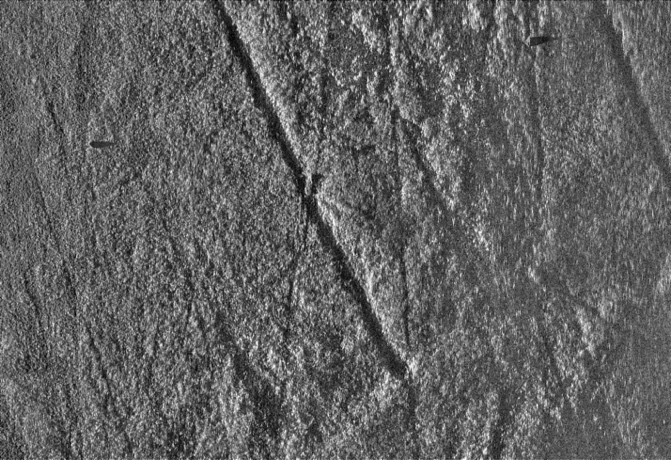


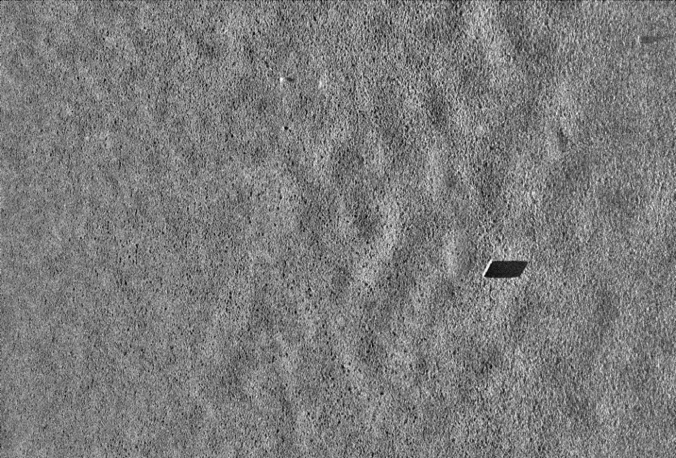

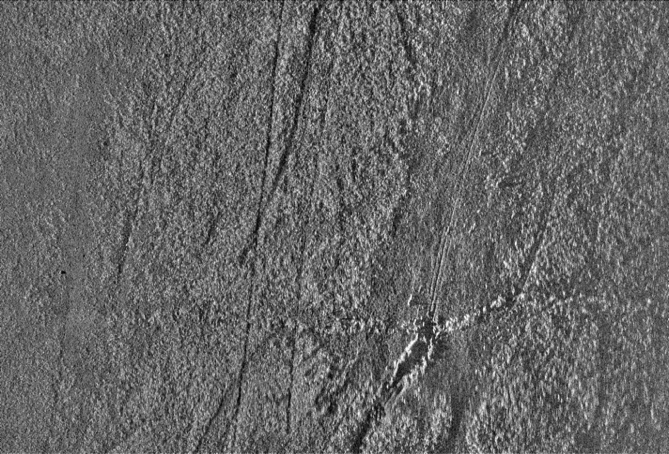


**Experimental Session Script – No Automation Block 1**

Now that you have completed training, you will examine 50 images that the unmanned submarine has collected of the sea floor along our proposed route. As mentioned before, it is important that all mines are detected; however, you cannot take too long with any one image because the mission needs to progress in a timely fashion. Looking at 50 images usually takes about 5 minutes and you will have a maximum of 8 minutes to examine them before we have to confirm our route to set sail. Therefore, please do not feel you have to rush for any particular image, but you will need to stay focused to make sure you get the task completed on time.

**Automation Training Script**

An Automated Target Recognition System has been designed to assist you to increase performance on the mine detection task. This system may help you make your decisions more quickly and help you detect more mines. The system uses computer vision to identify the presence of mines. The system isn’t entirely perfect. If we want it to detect more mines (in other words, if we want it to miss detecting very few mines), often it can make a false alarm mistake and say a mine is present when it really isn’t. The system designers can change the system to trade off how many mistakes of this type the automation makes.

Here is an example of what the Automated Target Recognition’s response may look like on one of the images. A rectangle will appear around a region on the sonar image where the system believes a mine is present. You will now have the opportunity to look at 4 different images from a previous mission that display the 4 possible responses that you may receive from the automated system. (*The experimenter now showed participants the images on the screen which were examples of a hit, miss, correct rejection and false alarm).*


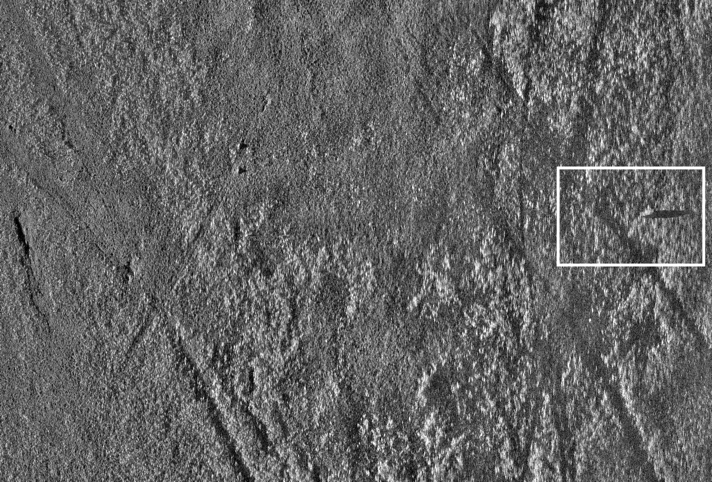


**Experimental Session Script – Automation Block 1**

Now that you have completed training on the automation, you can use the Automated Target Recognition System to help you with this block of trials. As discussed earlier, this system may help you make your decisions more quickly and help you detect more mines. The system uses computer vision to identify the presence of mines. If we want it to detect more mines (in other words, if we want it to miss detecting very few mines), often it can make a false alarm mistake and say a mine is present when it really isn’t. The system designers can change the system to trade off how many mistakes of this type the automation makes.

***For informed group only, High FA rate condition:***

For this mission, your commander has set the sensitivity of the device high. **That means that it is expected that 24% of the trials the automation is going to have a false alarm where it says a mine is present but in fact there is no mine. However, this also means that the system may detect more mines.**

***For informed group only, Low FA rate condition:***

For this mission, your commander has set the sensitivity of the device low. **That means that it is expected that 12% of the trials the automation is going to have a false alarm where it says a mine is present but in fact there is no mine. However, this also means that the system may miss a few more mines.**

You can use the automation to help inform your decision, but you must make the final decision. This mission block will consist of 100 images collected from the unmanned submarine. You will have a maximum of 15 minutes to examine the images before we have to confirm our route to set sail though most times it should take only about 10 minutes to look through 100 images. Therefore, please do not feel you have to rush for any particular image because it is important to detect mines, but you will need to stay on task so we can confirm our route.

**Experimental Session Script – No Automation Block 2**

For this mission we are re-calibrating the automated system so you will not have access to it. You will examine 50 images that the unmanned submarine has collected of the sea floor along our proposed route. As mentioned before, it is important that all mines are detected; however, you cannot take too long with any one image because the mission needs to progress in a timely fashion. Looking at 50 images usually takes about 5 minutes and you will have a maximum of 8 minutes to examine them before we have to confirm our route to set sail. Therefore, please do not feel you have to rush for any particular image, but you will need to stay on task.

**Experimental Session Script – Automation Block 2**

The Automated Target Recognition System has been recalibrated and can help you with this block of trials. As mentioned before, this system may help you make your decisions more quickly and help you detect more mines. The system uses computer vision to identify the presence of mines. The system isn’t entirely perfect. If we want it to detect more mines, often it can make mistakes and say a mine is present when it really isn’t. The system designers can change the system to trade off how many mistakes of this type the automation makes.

***For informed group only, High FA rate condition:***

During the recalibration, your commander has set the sensitivity of the device high for this mission. **That means that it is expected that 24% of the trials the automation is going to have a false alarm where it says a mine is present but in fact there is no mine. However, this also means that the system may detect more mines.**

***For informed group only, Low FA rate condition:***

During the recalibration, your commander has set the sensitivity of the device low for this mission. **That means that it is expected that 12% of the trials the automation is going to have a false alarm where it says a mine is present but in fact there is no mine. However, this also means that the system may miss a few more mines.**

You can use the automation to help inform your decision, but you must make the final decision. This mission block will consist of 100 images collected from the unmanned submarine. You will have a maximum of 15 minutes to examine the images before we have to confirm our route to set sail though most times it should take only about 10 minutes to look through 100 images. Therefore, please do not feel you have to rush for any particular image because it is important to detect mines, but you will need to stay on task so we can confirm our route.

**Response Time Results**

A significant difference was found between the response times when a mine was present for Automation Condition, *F*(1.74, 118.6) = 147.9, *p* < .01, *η_p_*^2^ = .685, where No Auto 1 showed a significantly longer response time compared to the other conditions (critical value = 0.51s) (A). No difference in response time was found between the three other conditions.

In the mine absent trials, a significant effect of response time was only found for Automation Condition, *F*(2.10, 142.5) = 68.8, *p* < .01, *η_p_*^2^ = .50, where similar to the trials when a mine was present, No Auto 1 showed a significantly longer response time compared to the other conditions (critical value = .56s) (B). Again, no difference in response time was found between the three other conditions.

Average response time of participants in the Informed (I) and Not Informed (NI) groups for each automation condition on trials where a mine was present (A) or absent (B).
